# Trajectories of depressive symptoms among community-dwelling Korean older adults: findings from the Korean longitudinal study of aging (2006–2016)

**DOI:** 10.1186/s12888-022-03905-3

**Published:** 2022-04-08

**Authors:** Jinhee Shin, Eunhee Cho

**Affiliations:** Mo-Im Kim Nursing Research Institute, Yonsei University College of Nursing, 606 Nursing Education Building, 50-1 Yonsei-ro, Seodaemoon-Gu, Seoul, 03722 Republic of Korea

**Keywords:** Aged, Depressive symptoms, Group-based trajectory modeling

## Abstract

**Background:**

Depression among older adults is an important public health concern associated with increased risk of suicide and decreased physical, cognitive, and social functioning. This study identified trajectories of depressive symptoms and investigated predictive variables of group-based trajectory modeling among Korean community-dwelling older adults.

**Methods:**

Participants comprised 2016 community-dwelling Korean adults over 65 years. Data from the years 2006–2016 of the Korean Longitudinal Study of Aging, a nationally representative panel survey that has been conducted every two years since 2006, were used. We employed a group-based trajectory modeling analysis to identify depressive symptom trajectories. Multinomial logistic regression analysis was conducted to identify predictors of each class of depressive symptoms.

**Results:**

Five depressive symptom trajectory groups were identified: Group 1, “None” (28.9%); Group 2, “Slowly worsening” (24.3%); Group 3, “Rapidly worsening” (17.5%); Group 4 “Improving” (12.4%); and Group 5, “Persistently severe” (16.9%). Older adults followed five distinct depressive symptom trajectories over 10 years. Mini-Mental State Examination scores, number of chronic diseases, educational level, and social activity were predictors associated with increasing depressive symptoms.

**Conclusions:**

This study showed that many older adults living in the community have depressive symptoms. To prevent and treat depression and aid successful mental health aging among older adults, the development of interventions should be tailored to target specific needs for each symptom trajectory. It is necessary to develop community-based interventions and strategies to identify and prevent depressive symptom trajectories among older adults.

## Background

Depression among older adults is an important public health concern associated with increased risk of suicide and decreased physical, cognitive, and social functioning [1]. Depression is a common psychiatric disorder that diminishes older people’s quality of life [2]. Specifically, it significantly reduces their health-related quality of life [3]. Studies have shown that the prevalence of major depression ranges from 0.9–9.4% for older adults living in the community, 14%–42% for those living in facilities [4]. Moreover, studies have suggested that many community-dwelling older adults aged 65 years and above (27%) experience depressive symptoms [5]. In South Korea, the proportion of patients with depression increased with age: 2.7% of individuals between 20 to 30 years, 5.7% between 40 to 50 years, 13.9% between 60 to 70 years, and 18.4% 80 years and older are diagnosed with depression [6]. Compared with younger individuals, older adults are at a significantly higher risk (HR 1.52, 95% CI 1.36–1.70) for suicide [6]. In South Korea, the suicide rate among older adults is the highest among Organization for Economic Co-operation and Development (OECD) countries, and depression among older adults is addressed as a public health concern. Doctors (41%) believe that depression is a common problem among older adults; however, they tend to complain of a variety of physical symptoms rather than reporting symptoms of depression. Thus, physical function blurs the depression diagnosis [7]. It was reported that 56% of the older adults who visited the geriatric center with physical symptoms were diagnosed with depression [8]. As older adults experience physical disability due to an increase in physical diseases and aging, they experience decreased self-esteem, making them vulnerable to depression [9]. For this reason, it is necessary to focus on depressive symptoms among older adults in South Korea.

Depressive symptoms experienced in older age have serious implications for health, functioning, and emotional distress. They are influenced by an individual’s subjective mood at the time of assessment and may vary according to one’s life course [10]. Depressive symptoms among older adults are associated with several negative outcomes, such as increased burden of disease [11], reduced abilities to cope with disease [12], poor quality of life [3], and early mortality [12]. Patterns of change in depressive symptoms can be influenced by a variety of risk factors, including biological and social factors, as well as individual, environment, and health behaviors [13]. Moreover, depressive symptoms have been associated with gender (female) [14], low income [15], low educational level [15], limited social support [16], functional impairment [16, 17], and physical diseases [16–18]. Predictors related to depression among older adults are associated with cognitive function [19, 20], exercise [21], and frequency of contact with family and friends [22]. The patterns of symptoms vary among individuals. Similar patterns can be observed for patients with consistently higher levels of depressive symptoms over time [23, 24]. A detailed longitudinal analysis not only shows how depression can appear over time, but also provides an opportunity to further understand the etiology. To this end, researchers have identified the trajectory of depressive symptoms during older adulthood and the potential risk factors associated with that trajectory [16, 17]. Depression may be cyclical, and individuals with depression may have periods when their symptoms subside. Among older adults, depression is heterogeneous in its severity, pattern, and progression, and functioning does not deteriorate in the same pattern or at the same rate for everyone [24]. When clarifying the causes of depression and improving treatment, it is important to see how different risk factors are related to different patterns of depressive symptoms. Most long-term studies of depressive disorders have been conducted with mixed-age participants in psychiatric inpatient or outpatient clinical settings [25]. The depressive trajectories among Mexican American and Taiwanese older adults have indicated that a disability in activities of daily living was the strongest predictor of depressive symptoms and needing social support [16, 26]. Depressive symptoms in many older adults are overlooked or untreated, resulting in a more serious form of disability. By identifying patterns of depressive symptoms among older adults in the community, depression can be diagnosed early and treated. Thus, an approach that considers different patterns of depressive symptoms could help policy makers and professionals to develop healthcare policies for older adults that consider individual characteristics and use limited healthcare resources more efficiently.

Multi-component collaborative intervention, through a collaboration of specialists, professional mental health care providers, and social networking, has the greatest impact on reducing depression [27]. Studies have shown that depression interventions included short-term screening for depression [28], a reduction in depressive symptoms [29], and a reduction in hospitalizations [30]. However, an intervention program that identifies and reflects long-term factors according to the trajectory of depression has not yet been examined. This study aimed to identify the trajectory of depressive symptoms among Korean older adults and predict factors related to it from a population-based sample in South Korea. The findings of this study will provide basic data to develop community-based intervention programs.

## Methods

### Data source and ethical concerns

The Korea Labor Institute collected data for the Korean Longitudinal Study of Aging (KLoSA) [31]. The KLoSA, a nationally representative multistage and stratified probability sample of community-dwelling adults, is a panel survey of Koreans who are 45 years or above. It is similar to the US Health and Retirement Study (HRS) [32]. The KLoSA recruited participants randomly using multilevel stratified sampling based on geographical areas and housing types representing the entire population of South Korea in 2006. Using the 2005 population and housing census data, sampling was conducted by classifying the population into 15 metropolitan cities and provinces. The sample was surveyed in each area according to administrative codes by applying the systematic extraction method. Each stage of sampling involved probability proportional to the size of the systematic sample of 2000 census enumeration districts (EDs) after stratification by the location and characteristic of a ED. The first phase included 10,254 individuals across 6,171 households (1.7 per household). All participants took part in a computer-assisted personal interview. The KLoSA has been conducted every two years since 2006 and is currently ongoing. The data used in this study are from 2006 (the first wave) to 2016 (the sixth wave). The KLoSA database is open to the public and anonymized data can be downloaded from the Korea Labor Institute website. The KLoSA was conducted after acquiring informed consent from the participants. The current study was conducted following the approval of the Institutional Review Board (Y-2020–0240).

### Participants

**Fig. 1:**
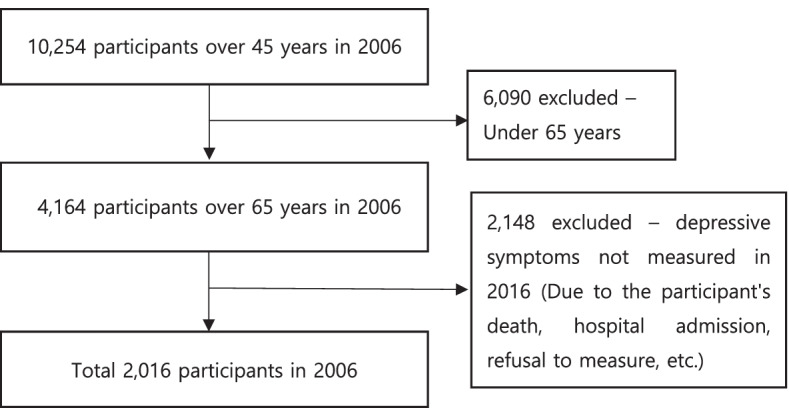
Participant selection process

In the baseline data from 2006, 10,254 individuals were surveyed, but we excluded 6,090 participants who were below 65 years. Attrition data can be heavily influenced by unnoticed outcomes of dropout cases, leading to potential misclassification of these cases into the GBTM [33]. In this study, we handled attrition as follows: Exclusion criteria were established when selecting participants for the first time. We followed the healthcare provider's suggestion and excluded dropout cases that were due to death and hospitalization. This is because the inclusion of these cases might lead to potential bias and misclassification in subgroup assignments as these individuals may be more likely to have depressive symptoms. Among the 4,164 participants selected from the baseline year, 2,148 were excluded due to death, hospitalization, or refusal to participate in 2016. The sample retention rate was 78.0% by the sixth wave. A total of 2,016 participants completed the survey of depressive symptoms continuously until 2016 (Fig. 1).

### Measures

#### Depressive symptoms

Depressive symptoms were measured using the Korean version of the Center for Epidemiologic Studies Depression Scale (CES-D10), which was developed in Boston [34]. The CES-D10 version was developed as a depressive symptoms screening test for epidemiological investigations. The scale consists of 10 items with scores ranging from 0 to 10, with higher scores indicating more severe depressive symptoms. For older adults, a score of four (sensitivity 100%, specificity 92%) was considered sufficient to diagnose clinically relevant depression [35, 36]. There is a high correlation between CES-D10 (a short version) and the original CES-D20 (comprising 20 items; *r* = 0.88, *p* < 0.001) with verified validity [34]. At the time of development, Cronbach’s alpha for CES-D10 was 0.88 [34]; for this study, it was 0.81.

#### Predictors of depressive symptom trajectories

Demonstrated risk factors in previous studies were selected as predictors of the trajectory of depressive symptoms. Demographic and socioeconomic factors included age, gender, existence of spouse, educational level, religion, current employment, and total household income. Educational level was divided into four categories (elementary school or lower, middle school, high school, and college or higher). Religion, existence of spouse, and current employment were measured as dichotomous variables (yes or no), and total household income and age were treated as continuous variables. For religion, respondents were instructed to indicate if they believed in any religion. A “yes” response indicated that the participant belonged to a religion while a “no” response indicated no affiliation with any religion.

Two tools were used to assess older people’s functional limitation: Korean activities of daily living (K-ADL) and Korean instrumental activities of daily living (K-IADL). The K-ADL assessed seven activities: bathing, dressing, eating, grooming, walking, transfers, and toileting [37]. For each activity, the participants received a score of 0 or 1 for their ability or the lack thereof, respectively. The K-ADL composite score ranged from 0 to 7, where 0 indicates high independence and 7 indicates high dependence. The K-IADL assessed 10 activities: grooming, housekeeping, preparing food, doing laundry, using transportation, handling money, phone use, shopping, traveling for a short distance, and taking medications [38]. For each activity, the participants received a score of 1 or 0 for their ability or the lack thereof, respectively. The K-IADL total scores ranged from 0–10, with higher scores indicating higher dependence; the construct was analyzed as a continuous variable.

Physical health was assessed as the total number of physician‐diagnosed chronic diseases (e.g., arthritis, hypertension, heart disease, cerebrovascular disease, cancer, chronic lung disease, diabetes, and psychiatric disorder), with the total count ranging from 0 to 8. Body Mass Index (BMI) was calculated as body weight (kg) divided by squared height (m^2^). Cognitive function was measured using the Korean-Mini-Mental Status Examination (K-MMSE); total scores ranged from 0 to 30, with higher scores indicating better cognitive function [39]. Cronbach’s alpha coefficient for K-MMSE at the time of development was 0.84 [40]; for this study, it was 0.79. Health behavior was measured through exercise at least once a week with response options of “yes” or “no”.

Social interaction was measured by the frequency of regular social activity (alumni/birthplace, religious, leisure/culture/sports, social, volunteer service, civic organizations/political party/interest groups) and contact with children (telephone, email, etc.). Responses were measured on a 9-point scale (1 = almost every day to 10 = not in contact). For analysis, the frequencies of social activity and contact with children were divided into five categories (almost daily, 2–3 times a week, 1–4 times a month, 1–6 times annually, and none).

### Statistical analysis

GBTM according to CES-D10 scores was used to identify trajectories of depressive symptoms during the follow-up period. GBTM was developed to identify different homogeneous subgroups with similar growth trajectories within longitudinal data of heterogeneous groups [41, 42]. Based on the nonparametric model by Heckman and Singer [43], the grouped-based trajectory model provided a simplified approach of uncovering hidden heterogeneity in the outcome trajectories among and between subjects [41, 42]. Unlike the general growth mixture model, the GBTM assumes initial homogeneity of the trajectory group membership and instead assigns the subject a probability of belonging to each latent group, thus being a simplified alternative [42]. GBTM is a special application of finite mixed modeling to identify clusters of individuals following a similar progression of specific outcomes over time [42]. The primary GBTM assumed data to be missing at random to allow for the probability of group membership assignment and attrition to be independent of biased approximations of trajectory group size [42]. In the enhanced model, attrition was modeled concurrently using logit distribution with the trajectory group as a function of time before dropping out. The approximation of the probabilities of dropping out are particular to the trajectory groups.

The final number of trajectories for this study was identified based on the smallest Bayesian information criterion (BIC), sample size adjusted BIC (SSABIC) [44], which is used to reduce the risk of overfitting the model to a single sample (lower values indicate a better model fit), and the Lo-Mendell-Rubin Likelihood Ratio Test (LMR-LRT), Adjusted Lo-Mendell-Rubin Likelihood Ratio Test (Adj. LMR-LRT), and Bootstrapped Likelihood Ratio Test (BLRT), which compare two adjacent class models (significant p-values indicate a better fit of the k class model compared to the k-1 class model). We further considered entropy values (0.40, 0.60, and 0.80 represent low, medium, and high class separation, respectively) [45, 46], sample size of the smallest class, and interpretability of each class trajectory. After determining group membership, using descriptive statistics based on the trajectory groups, sample characteristics from the 2006 data were described and compared. Finally, multinomial logistic regression was used to predict depressive symptoms trajectory group cluster associations as a function of the 2006 sample characteristics. STATA, version 16/SE (Stata Corp., College Station, Texas) was used for data analysis (TRAJ, BLRT, Adj. LMR-LRT, and multinomial logistics regression).

## Results

### Depressive symptom trajectories

**Fig. 2:**
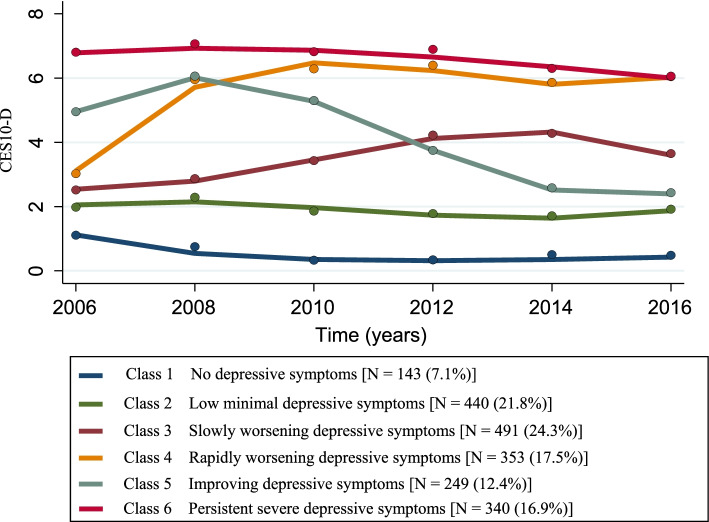
Depressive symptom trajectories over 10 years in community-dwelling Korean older adults

The first trajectory group comprised 7.1% of the sample (*n* = 143) and was labeled as “No depressive symptoms” (Class 1). The second trajectory group (Class 2) comprised 21.8% (*n* = 440) of the sample and was labeled as “Persistent minimal depressive symptoms.” The Classes 1 and 2 showed no clinical depressive symptoms at baseline and consistently showed no clinical depressive symptoms after 10 years. The third and largest trajectory group comprised 24.3% of the sample (*n* = 491) and was labeled as the “Slowly worsening depressive symptoms” (Class 3). This trajectory group showed no depressive symptoms at baseline; the depressive symptoms increased slowly and worsened to a clinical level after six years. The fourth trajectory group (Class 4) comprised 17.5% (*n* = 353) of the sample and was labeled as “Rapidly worsening depressive symptoms.” This trajectory group showed no depressive symptoms at baseline, but depressive symptoms started increasing after 6 years till 10 years. The fifth trajectory group (Class 5) comprised 12.4% (*n* = 249) of the sample and was labeled as “Improving depressive symptoms.” This trajectory group showed depressive symptoms at baseline, but no depressive symptoms were present after 10 years. The final trajectory group (Class 6) comprised 16.9% (*n* = 340) of the sample and was labeled as “Persistently severe depressive symptoms.” This trajectory group showed depressive symptoms at baseline and the symptoms were maintained over time (Fig. 2).

Next, lower values of the BIC and SSABIC indicated a better fit of the model. The LMR-LRT, and Adj. LMR-LRT and BLRT compared two adjacent class models and revealed a significant p-value, indicating a better fit of the k class model compared to the k-1 class model. In using this model guide, we further considered entropy values. Most of the models had an entropy value of 0.6 or higher. In this study, we considered an entropy value of 0.6 (medium) or higher [45, 46]. We also considered the sample size of the smallest class (< 5%) in developing the model based on the LMR-LRT and the Adj. LMR-LRT and BLRT. Most of the model fit indices suggested that the 5- and 6-class models were the optimal (e.g., lower BIC and SSABIC values, and statistically significant p-values for LMR-LRT, Adj. LMR-LRT, and BLRT). The 6-class model (BIC = -27,199.58; SSABIC = -27,226.45) had better fit indices compared to the 5-class model (BIC = -27,204.47; SSABIC = -27,226.86), and the former had each class of an acceptable size. Thus, we rejected the 5-class model in favor of the 6-class model. The 7-class model was not statistically significant with respect to its Adj. LMR-LRT *p*-value. Table 1 shows the model fit for all trajectories and the percentage of participants in each trajectory.

**Table 1:** Model fit and percentage of older adults in trajectories

Fit statistics	2 classes	3 classes	4 classes	5 classes	**6 classes**^a^	7 classes
LL	-27,508.12	-27,230.09	-27,198.61	-27,109.36	**-27,085.44**	-27,071.06
BIC	-27,546.16	-27,287.15	-27,274.70	-27,204.47	**-27,199.58**	-27,204.21
SSABIC	-27,555.12	-27,300.59	-27,292.62	-27,226.86	**-27,226.45**	-27,235.57
Entropy	0.785	0.755	0.663	0.660	**0.624**	0.638
LMR-LRT *p-value*	< 0.001	< 0.001	< 0.001	< 0.001	** < 0.001**	< 0.001
Adj. LMR-LRT *p-value*	< 0.001	< 0.001	< 0.001	< 0.001	** < 0.001**	0.084
BLRT *p-value*	< 0.001	< 0.001	< 0.001	< 0.001	** < 0.001**	< 0.001
Class %						
Class 1	41.99	15.58	14.71	13.45	7.54	8.39
Class 2	58.01	45.40	41.88	31.97	23.53	23.49
Class 3		39.02	19.57	12.01	20.71	18.82
Class 4			23.84	23.32	18.13	1.99
Class 5				19.26	13.25	16.41
Class 6					16.84	14.33
Class 7						16.58

*N* = 2016, *LL* Log-Likelihood value, *BIC* Bayesian information criterion, *LMR-LRT* Lo-Mendell-Rubin Likelihood Ratio Test, *Adj. LMR-LRT* Adjusted Lo-Mendell-Rubin Likelihood Ratio Test, *BLRT* Bootstrapped Likelihood Ratio Test

^a^Selected class solution is in bold

### Reclassification of depressive symptom trajectories

The six classes of GBTM were reclassified into five trajectory groups according to the clinical criteria for depressive symptoms. Clinically, the cutoff point of depressive symptoms is CES-D10 ≥ 4 [35]. Class 1 and 2 trajectories were groups with no clinical depressive symptoms, while the other trajectories showed some form of depressive symptoms over the course of 10 years. The model was reclassified into the following five trajectory groups based on the occurrence of depressive symptoms: Group 1 (Classes 1 and 2) – “None”; Group 2 (Class 3) – “Slowly worsening”; Group 3 (Class 4) – “Rapidly worsening”; Group 4 (Class 5) – “Improving”; and Group 5 (Class 6) – “Persistently severe” (Table 2).

**Table 2:** Reclassification of depressive symptom trajectories

Original trajectories	N(%)	Reclassified trajectories	N(%)
Class 1 No depressive symptoms	143(7.1)	Group 1 None	583(28.9)
Class 2 Persistent minimal depressive symptoms	440(21.8)		
Class 3 Slowly worsening depressive symptoms	491(24.3)	Group 2 Slowly worsening	491(24.3)
Class 4 Rapidly worsening depressive symptoms	353(17.5)	Group 3 Rapidly worsening	353(17.5)
Class 5 Improving depressive symptoms	249(12.4)	Group 4 Improving	249(12.4)
Class 6 Persistently severe depressive symptoms	340(16.9)	Group 5 Persistently severe	340(16.9)

### Participant characteristics

Table 3 shows the general baseline characteristics of participants according to the five trajectory groups. At baseline, the mean participant age was 70.96 years (SD = 4.87), and 56.2% were female. Most (71.3%) had elementary school or lower education and 69.7% had spouses. A total of 23.1% of the participants were employed and had an average of 1.01 (SD = 1.01) chronic diseases. The mean K-ADL score was 0.12 (SD = 0.72), the mean K-IADL score was 0.52 (SD = 1.57), and the mean K-MMSE score was 23.80 (SD = 5.35). The mean BMI score was 23.10 (SD = 2.82), and 66.3% exercised at least once a week.

**Table 3:** Baseline characteristics of older adults according to depressive symptom trajectory groups (*N* = 2,016)

Variables	Total (*n* = 2,016)	Depressive symptom trajectories					
		**Group 1 None (*n* = 583)**	**Group 2 Slowly worsening (*n* = 491)**	**Group 3 Rapidly worsening (*n* = 353)**	**Group 4 Improving (*n* = 249)**	**Group 5 Persistently severe (*n* = 340)**	***p***
Age, mean	70.96 ± 4.87	70.19 ± 4.82	70.71 ± 4.76	71.71 ± 4.90	71.15 ± 4.95	71.74 ± 4.78	< .001
Gender, n (%)							
Male	815(43.8)	308(52.8)	215(43.8)	129(36.5)	79(31.7)	84(24.7)	< .001
Female	1201(56.2)	275(47.2)	276(56.2)	224(63.5)	170(68.3)	256(75.3)	
Education level, n (%)							
Elementary or lower	1437(71.3)	343(58.8)	341(69.5)	253(71.7)	210(84.8)	290(85.3)	< .001
Middle school	206(10.2)	72(12.3)	61(12.4)	36(10.2)	15(6.0)	22(6.5)	
High school	260(12.9)	102(17.5)	62(12.6)	58(15.3)	19(7.6)	23(6.8)	
College or higher	113(5.6)	66(11.3)	25(5.5)	6(2.8)	5(2.0)	5(1.5)	
Have a religion, n (%)							
Yes	1132(56.2)	323(55.9)	291(59.3)	196(55.5)	132(53.0)	153(45.0)	.527
No	884(43.8)	257(44.1)	200(40.7)	157(44.5)	117(47.0)	153(55.0)	
Have a spouse, n (%)							
Yes	1405(69.7)	453(77.7)	363(73.9)	232(65.7)	163(65.5)	194(57.1)	< .001
No	611(30.3)	130(22.3)	128(26.1)	121(34.3)	86(34.5)	146(42.9)	
Current job							
Yes	466(23.1)	162(27.8)	138(28.1)	70(19.8)	55(22.1)	41(12.1)	< .001
No	1550(76.9)	421(72.2)	353(71.9)	283(80.2)	194(77.9)	299(87.9)	
Total household income (US$), mean^a^	16,525.5 ± 21,997.5	17,208.8 ± 22,857.0	16,151.8 ± 22,972.7	15,001.5 ± 17,515.4	16,553.5 ± 20,063.5	17,455.6 ± 24,477.2	.557
K-ADL score, mean	0.12 ± 0.72	0.02 ± 0.31	0.05 ± 0.45	0.08 ± 0.55	0.22 ± 0.93	0.35 ± 1.28	< .001
K-IADL sore, mean	0.52 ± 1.57	0.27 ± 0.85	0.36 ± 1.27	0.49 ± 1.42	0.84 ± 2.10	1.00 ± 2.29	< .001
K-MMSE, mean	23.80 ± 5.35	25.30 ± 4.39	24.53 ± 4.71	23.19 ± 5.86	23.30 ± 5.71	21.15 ± 5.75	< .001
Number of chronic diseases, mean	1.01 ± 1.01	0.83 ± 0.91	0.87 ± 0.90	1.07 ± 1.00	1.11 ± 1.05	1.38 ± 1.17	< .001
BMI, mean	23.10 ± 2.82	23.29 ± 2.66	23.00 ± 2.87	23.12 ± 2.82	22.90 ± 2.88	23.04 ± 2.94	.320
Exercise at least once a week, n (%)							
Yes	1337(66.3)	354(60.7)	305(62.1)	229(64.9)	184(73.9)	256(77.9)	< .001
No	679(33.7)	229(39.3)	186(37.9	124(35.1)	65(26.1)	75(22.1)	
Frequency of contact with children, n (%)							
Almost daily	385(19.1)	123(21.1)	80(16.3)	36(17.8)	50(20.1)	69(20.3)	.414
2–3 times a week	1007(50.0)	294(50.4)	259(52.7)	185(52.4)	112(45.0)	157(46.2)	
1–4 times a month	513(25.4)	137(23.5)	125(25.5)	86(24.4)	74(29.7)	91(26.8)	
1–6 times annually	99(4.9)	26(4.5)	22(4.5)	16(4.5)	13(5.2)	22(6.5)	
None	12(0.6)	3(0.5)	5(1.0)	3(0.8)	0(0.0)	1(0.3)	
Frequency of regular social activity, n (%)							
Almost daily	209(10.4)	70(12.0)	58(11.8)	18(5.1)	38(15.3)	25(7.4)	< .001
2–3 times a week	513(25.4)	150(25.7)	119(24.2)	96(27.2)	51(20.5)	97(28.5)	
1–4 times a month	147(7.3)	29(5.0)	27(5.5)	33(9.3)	18(7.2)	40(11.8)	
1–6 times annually	648(32.1)	188(32.2)	144(29.3)	111(31.4)	94(37.8)	111(32.6)	
None	499(24.8)	146(25.0)	143(29.1)	95(26.9)	48(19.3)	67(19.7)	
CES-D10 score, mean	3.44 ± 2.77	1.71 ± 1.83	2.52 ± 2.08	2.45 ± 1.38	5.33 ± 2.31	7.37 ± 1.46	< .001

*K-ADL* Korean Activities of Daily Living, *K-IADL* Korean Instrumental Activities of Daily Living, *K-MMSE* Korean-Mini-Mental Status Examination, *BMI* Body Mass Index, *CES-D10* Center for Epidemiologic Studies Depression Scale

^a^Average exchange rate in 2006: 1000 Korean won/US$

The five trajectory groups differed significantly in age, gender, education level, existence of spouse, employment, K-ADL, K-IADL, K-MMSE, number of chronic diseases, exercise patterns, frequency of social activity, and CES-D10. The highest average baseline age (mean = 71.74), females (75.3%), and those with elementary or lower education (85.3%) were found in the “Persistently severe” trajectory group. In contrast, the lowest average baseline age (mean = 70.19), females (47.2%), and those with elementary or lower education (58.8%) were found in the “None” trajectory group. The highest and lowest values of existence of a spouse were found in the “None” (77.7%) and “Persistently severe” trajectory groups (57.1%), respectively. The average scores for K-ADL at 0.35 (SD = 1.28) and K-IADL at 1.00 (SD = 2.29) were highest in the “Persistently severe” trajectory group. The K-MMSE score was the highest in the “None” trajectory group at 25.03 (SD = 4.39). For the frequency of regular social activities, the highest score for meeting almost daily was found in the “Improving” trajectory group (15.3%), while the lowest was found in the “Rapidly worsening” trajectory group (5.1%).

### Predictors of depressive symptom trajectory groups

Table 4 shows the results of the subgroup analysis using multinomial logistic regression to predict variables associated with trajectory groups of depressive symptoms at baseline. In the final model, the multicollinearity diagnostic test showed variance inflation factor values of 1.01 to 2.10, confirming that there was no problem with multicollinearity. In the multinomial logistic regression model, we adjusted for age, gender, education level, religion, spouse, job, and income. Baseline chronic disease count, K-MMSE, and regular social activity were associated with a higher relative risk of being in two depressive trajectory groups—"Rapidly worsening" and “Improving.” Compared to the "None" trajectory group, participants in the "Rapidly worsening" trajectory group were at a relatively higher risk of an increased number of chronic diseases (relative risk [RR] = 0.25, *p* = 0.001). The "Rapidly worsening " trajectory group was associated with decreased K-MMSE scores (RR = -0.05, *p* = 0.005). Furthermore, compared with participants engaging in social activities almost daily, the frequency of regular social activities was associated with increased depressive symptoms: 2–3 times a week (β = 0.81, *p* = 0.14), 1–4 times a month (β = 1.41, *p* = 0.009), 1–6 times annually (β = 0.85, *p* < 0.001), and none (β = 0.76, *p* = 0.005). Compared to the "Persistently severe " group, the “Improving trajectory” group was more likely to have standard K-MMSE scores (RR = 0.07, *p* < 0.001) and fewer chronic diseases (RR = -0.25, *p* = 0.005). Moreover, compared with those who engaged in social activities almost daily, the frequency of regular social activities decreased the relative risk significantly: 2–3 times a week (RR = -1.17, *p* = 0.047), 1–4 times a month (RR = -1.16, *p* =  < 0.001), 1–6 times annually (RR = -0.73, *p* = 0.004), and none (RR = -0.67, *p* = 0.019).

**Table 4:** Multinomial logistic regression predicting depressive symptom trajectory groups (*N* = 2,016)

Variables	None (Group 1) vs Slowly worsening (Group 2)			None (Group 1) vs Rapidly worsening (Group 3)			Persistently severe (Group 5) vs Improving (Group 4)		
	**RR**	**95% CI**	***P***	**RR**	**95% CI**	***P***	**RR**	**95% CI**	***P***
Age	0.02	-0.01, 0.05	.130	**0.04**	**0.01, 0.07**	**.017**	0.01	-0.03, 0.05	.649
Gender (ref. male)	0.18	-0.14, 0.50	.274	0.23	0.14, 0.60	.700	0.09	-0.03, 0.56	.700
Education level (ref. Elementary or lower)									
Middle school	-0.10	-0.50, 0.30	.611	-0.05	-0.53, 0.42	.822	-0.39	-1.11, 0.32	.281
High school	-0.35	-0.76, 0.05	.085	0.30	-0.13, 0.73	.177	-0.32	-1.02, 0.37	.362
College or higher	**-1.00**	**-1.55, -0.45**	** < .001**	**-1.78**	**-2.68, -0.88**	** < .001**	-0.07	-1.39, 1.24	.913
Have a religion (ref. No)	0.10	-0.17, 0.36	.469	-0.13	-0.43, 0.17	.396	-0.06	-0.42, 0.30	.746
Have a spouse (ref. Yes)	-0.00	-0.33, 0.33	.985	0.22	-0.13, 0.58	.220	-0.23	-0.63, -0.17	.251
Current job (ref. Yes)	-0.12	-0.43, 0.19	.439	0.25	-0.19, 0.61	.185	**-0.64**	**-1.13, -0.15**	**.010**
Total household income	-0.00	-0.00, 0.00	.510	-0.00	-0.00, 0.00	.227	-0.01	-0.00, 0.00	.414
K-ADL score	0.32	-0.17, 0.81	.203	0.24	-0.25, 0.73	.346	-0.04	-0.33, 0.25	.768
K-IADL sore	-0.03	-0.18, 0.12	.694	0.03	-0.12, 0.18	.671	0.07	-0.75, 0.21	.359
K-MMSE	-0.01	-0.04, 0.02	.457	**-0.05**	**-0.08, -0.01**	**.005**	**0.07**	**-0.03, 0.10**	** < .001**
Number of chronic diseases	0.06	-0.08, 0.21	.401	**0.25**	**0.10, 0.41**	**.001**	**-0.25**	**-0.04, -0.07**	**.005**
BMI	-0.04	-0.08, 0.01	.142	-0.04	-0.09, 0.01	.142	-0.01	-0.06, 0.07	.942
Exercise at least once a week (ref. No)	-0.20	-0.48, 0.09	.170	-0.14	-0.46, 0.19	.405	-0.26	-0.69, 0.17	.232
Frequency of contact with children (ref. Almost daily)									
2–3 times a week	-0.06	-0.51, 0.38	.183	0.12	-0.26, 0.50	.539	-0.07	-0.54, 0.39	.753
1–4 times a month	0.12	0.54, 0.77	.168	0.15	-0.29, 0.58	.509	-0.04	-0.55, 0.47	.877
1–6 times annually	-0.07	-0.50, 0.37	.345	0.14	-0.63, 0.90	.724	-0.29	-0.10, 0.51	.476
None	0.10	-0.34, 0.54	.358	0.70	-1.05, 2.46	.431	-13.23	-1602.99, 1576.53	.987
Frequency of regular social activity (ref. Almost daily)									
2–3 times a week	0.23	-0.11, 0.58	.664	**0.81**	**0.20, 1.41**	**.014**	**-1.17**	**-1.81, -0.52**	**.047**
1–4 times a month	0.27	-0.12, 0.66	.776	**1.41**	**0.65, 2.17**	**.009**	**-1.16**	**-1.95, -0.34**	** < .001**
1–6 times annually	0.33	-0.35, 1.01	.731	**0.85**	**0.26, 1.44**	** < .001**	**-0.73**	**-1.33, -0.12**	**.004**
None	0.73	-0.83, 2.30	.761	**0.76**	**0.16, 1.37**	**.005**	**-0.67**	**-1.33, -0.00**	**.019**

*K-ADL* Korean Activities of Daily Living, *K-IADL* Korean Instrumental Activities of Daily Living, *K-MMSE* Korean-Mini-Mental Status Examination, *BMI* Body Mass Index, *CES-D10* Center for Epidemiologic Studies Depression Scale, *RR* relative risk

The relative risk of being on the “Slowly worsening” trajectory was associated with education level (college or higher; RR = -1.00, *p* =  < 0.001). The relative risk of being on the “Rapidly worsening” trajectory increased with age (RR = 0.04, *p* = 0.017), and decreased with education level (college or higher; RR = -1.78, *p* =  < 0.001). Having a current job (RR = 0.64, *p* = 0.010) was associated with a higher relative likelihood of being on the “Improving” trajectory.

## Discussion

This was a population-based study on the long-term trajectory of depressive symptoms among older adults living in nationally represented communities in South Korea. Depression is an important mental health concern among older adults. In previous studies, depressive symptoms were identified as a strong predictor of depression [47]. We identified five trajectory groups of depressive symptoms among community-dwelling older adults using data from a national Korean population-based survey over a 10-year period. In a previous study that identified four trajectories of middle-aged and older adults’ depression in the United States, depression status was reported as never (85.8%), increasing (6.3%), decreasing (3.2%), and persistently moderate/high (4.7%) [48]. Further, in one study, the US national data (HRS) was used to identify six trajectories of depression symptoms using the eight-item CES-D over 11 years; the trajectories were as follows: minimal depressive symptoms (15.9%), low depressive symptoms (36.3%), moderate and stable depressive symptoms (29.2%), high but decreasing depressive symptoms (6.6%), moderate but increasing depressive symptoms (8.3%), and persistently high depressive symptoms (3.6%) [18]. Our findings show that older adults living in communities in South Korea have more depressive symptoms than those in the United States. These results are consistent with previous reports indicating that depressive symptoms and depression among community-dwelling older adults are a natural, chronic, and unremitting process [48].

By confirming the trajectories of depressive symptoms and risk factors, many older adults can be treated for the depressive symptoms that are identified, rather than neglected. We suggest that each depressive symptom trajectory found over the course of 10 years could be useful in predicting and more accurately distinguishing the related risk factors. Over time, compared with those in the "None" trajectory, those in the "Rapidly worsening" trajectory had a lower educational level, decreased K-MMSE scores, an increased number of chronic diseases, and decreased regular social activities. Furthermore, compared with the "Persistently severe" trajectory group, those in the "Improving" trajectory group had a job, increased K-MMSE scores, a decreased number of chronic diseases, and increased regular social activities, which were related to a decrease in the risk of depressive symptoms. Common predictors of the two trajectories ("Persistently severe" and "Improving") were identified by K-MMSE scores, the number of chronic diseases, and frequency of social activity. In previous studies, lower levels of education were associated with depressive disorders [49], whereas higher levels of education were associated with reduced depressive disorders [50, 51]. Additionally, employment status was associated with depression, and unemployment significantly increased the odds of having depression [52]. Our study found that participants with higher levels of education had a relatively lower risk of being in the "Rapidly worsening " trajectory; having college or higher-level education at baseline was associated with a decrease in the risk of depressive symptoms.

Unemployment status was associated with a higher relative risk of being “persistently severe” than “improving” in the trajectory group. In Europe and the USA, unemployed older adults had a higher incidence rate for depression [53]. Older adults that are unemployed are more likely to have high levels of depression [54] because of low income levels and poor quality of life [55]. Having a job could further influence or contribute to them having good social interaction, psychological well-being, and improved quality of life [54]. Despite being able to work longer in the Korean society, Korean culture requires people to retire when they reach 65 years. Therefore, it is necessary to create jobs where older adults can work, even after they reach the age of retirement. Furthermore, it is necessary to provide opportunities for social interaction and communication for older adults who cannot get jobs due to their health conditions.

In the present study, there was a relationship between depressive symptoms and cognitive function; participants with depressive symptoms were more likely to show a decrease in cognitive function scores (K-MMSE), while reduced depressive symptoms were associated with an increase in cognitive function scores. One study found that participants with depressive symptoms were at high risk for dementia [56]. Whether depression is a cause of dementia or simply increases its likelihood has not been determined; however, the link between depression and the risk of cognitive decline suggests a strong positive association [57]. There is an association between the severity of depression among older adults and episodic memory, executive functioning, and information processing speed [58]. Older adults with depression have more cerebral white matter and other subcortical abnormalities compared with healthy older adults [59]. Frequent and chronic long-term depression impart impairing effects on brain function and debase human capital [60]. Individuals with a greater severity of depressive symptoms are more likely to present cognitive impairment [60], and those with high and rapidly increasing depressive symptoms are at risk for dementia [56]. Hence, older adults with depression require regular screening and evaluation of cognitive functions by clinicians.

Previous studies have suggested that chronic diseases are associated with an increased risk of depressive symptoms [61]. Older adults with consistently low health status with respect to chronic diseases were more likely to belong to the depressive symptom trajectory; an increase in the number of chronic diseases predicted a significant increase in depressive symptoms, and vice versa. The chronic disease process causes difficulties related to symptoms, the treatment process, disruptions in family relationships, loss of certain abilities, and changes in body image [62]. A high prevalence of depression has been reported among patients with a variety of chronic conditions over the past few decades, demonstrating a close relationship between chronic disease and depression [62]. Chronic disease and depression are linked in many ways because physical and mental health influence each other. Chronic disease can also limit social activities due to difficulties in physical activity [62], which worsen depression [4]. The present study found that the frequency of regular social activities at baseline was associated with decreased risk of depressive symptoms. In the relationship between social support and depression among older adults, having fewer social relationships has been suggested as a predictor of depression [4]. Depression fundamentally changes one's perceptions and interactions with the surrounding environment, affecting the social environment [60]. In a previous study examining social support and depression among older Asians, larger social networks, more contact with family and friends, family support, and social support were associated with reduced depressive symptoms among community-dwelling older adults [63]. Social support that may help improve depression is also a modifiable factor in preventing cognitive decline [4, 56]. Our findings also suggest that social activities are important for reducing depressive symptoms. Furthermore, our findings suggest a need for concrete intervention plans to help older adults maintain close social relationships with those around them who can provide ongoing chronic disease management and psychological support, to prevent and alleviate depressive symptoms.

Successful healthy aging is defined as reaching older adulthood with the absence of major disease, depression, or disability; high cognitive and physical function; active social engagement; and satisfaction with life [64]. Individual components of successful aging are also components of well-established risk factors, and our results confirm that depressive symptoms among older adults are closely associated with and influence the conditions for successful aging. Many intervention studies have shown that programs (e.g., exercise, life review therapy, cognitive improvement, and social support networks) [65–67] to reduce depressive symptoms among community-dwelling older adults are effective. Community health care providers can employ a chronic condition management approach to depression, enabling intensive management of at-risk individuals, regular at-home visits, communication for cooperation between home care and primary care providers, and collaboration referral to specialized mental health services [68]. It is necessary to develop community-based interventions and strategies to identify depressive trajectory symptoms among older adults, manage predictive factors in the community, and develop interventions and strategies to manage or prevent them.

There were several limitations to this study. First, our study targeted older adults living in the community and did not include those who were admitted to nursing homes and hospitals. Second, it was not possible to fully assess whether participants had been diagnosed with depression and treated with medication. Third, participants were asked to state if they engaged in exercise at least once a week; however, this may not have effectively indicated the level of exercise needed for the prevention of diseases among older adult populations. Finally, the study could not account for substantial classification uncertainty. Therefore, there may be bias in estimating the associations between subgroup membership and secondary variables such as education, MMSE, and ADL. Despite these limitations, our study is significant, as it used a representative sample for longitudinal follow-up. In addition, by identifying risk factors affecting CES-D10 scores, the results can be utilized to prevent depression among older adults in the future.

## Conclusions

Given the rapidly aging populations globally, it is likely that the number of older adults with depressive symptoms will continue to increase. Our findings showed that community-dwelling Korean older adults followed five distinct depressive symptom trajectories over 10 years. Our findings indicated that low educational level, no current employment, decreased K-MMSE scores, more chronic diseases, and low frequency of regular social activities were related to an increased risk of depressive symptoms among community-dwelling Korean older adults. Health care providers should periodically screen older adults for depressive symptoms, and interventions should be developed to address individual risk factors. Depression management and prevention programs by local health managers should be applied through screening in clinics and hospitals for older adults belonging to trajectories that may be headed toward increased depression. Health care providers should consider our findings to prevent depressive symptoms among older adults. Furthermore, given that the suicide rate among older adults in South Korea is the highest among OECD countries, we recommend that policy makers and other relevant stakeholders establish a national monitoring system for depression among older adults.

## Data Availability

The data from the KLoSA are publicly available for download on the KLoSA website (https://survey.keis.or.kr/klosa/klosa01.jsp).

## Abbreviations

BIC: Bayesian information criterion
BLRT: Bootstrapped Likelihood Ratio Test
BMI: Body mass index
CES‐D10: Center for Epidemiologic Studies Depression Scale
GBTM: Group-based trajectory modeling
LMR-LRT: Lo-Mendell-Rubin Likelihood Ratio Test
K-ADL: Korean Activities of Daily Living
K-IADL: Korean Instrumental Activities of Daily Living
KLoSA: Korean Longitudinal Study of Aging
K-MMSE: Korean-Mini-Mental Status Examination
OECD: Organization for Economic Co-operation and Development
SSABIC: Sample size adjusted BIC
